# Marginal Sinus and Bleeding in Women with a Low-Positioned Placenta: A Narrative Synthesis Systematic Review

**DOI:** 10.1089/whr.2025.0002

**Published:** 2025-06-02

**Authors:** Elisabetta Colciago, Anna Locatelli, Simona Fumagalli, Valeria Poletti De Chaurand, Federica Fernicola, Antonella Nespoli, Sara Ornaghi

**Affiliations:** ^1^ Department of Medicine and Surgery, University of Milano-Bicocca, Milan, Italy.; ^2^ Obstetrics Unit, IRCCS San Gerardo dei Tintori, Monza, MB, Italy.

**Keywords:** low-positioned placenta, marginal sinus, antepartum hemorrhage, postpartum hemorrhage, bleeding risk

## Abstract

**Objective:**

To ascertain the impact of marginal sinus on the risk of antepartum, intrapartum, and postpartum hemorrhage in women with a low-positioned placenta.

**Data Sources:**

PubMed, Scopus, EMBASE, and the Cochrane Library databases (1980–2024).

**Study Selection:**

Systematic reviews and quantitative primary research studies reporting a diagnosis of low-positioned placenta with the presence or absence of marginal sinus. Outcome measures: antepartum, intrapartum, and postpartum hemorrhage.

**Data Extraction and Synthesis:**

Of the 8140 articles screened for eligibility, 171 were sought for full-text review, and 6 were included for analysis. The systematic review comprises six cohort studies, two prospective and four retrospective, for a total of 621 women with a low-positioned placenta. Five studies assessed the impact of marginal sinus on antenatal hemorrhage, two examined its influence on intrapartum hemorrhage, and one study also evaluated postpartum hemorrhage.

**Conclusions:**

The studies displayed adequate representativeness of exposed individuals. Limitations included retrospective design with a small sample size, different gestational ages at diagnosis of low-positioned placenta, and substantial heterogeneity in outcomes.

**Abstract:**

Among the five studies examining the relationship between marginal sinus and antepartum bleeding, a significant association was reported in four, while one found no such link. The only two studies examining the relationship between marginal sinus and intrapartum hemorrhage reported no association. Additionally, one of these studies identified lower rates of postpartum bleeding in women with normally located placental tissue and a marginal sinus reaching the internal os, compared with women with low-positioned placental tissue. The risk of bleeding in women with a low-positioned placenta and marginal sinus is still poorly evaluated. The evidence from the included studies lacked consistency and conclusive findings, highlighting the need for further research to elucidate this association and inform clinical management effectively. Additionally, studies failed to address the significance of marginal sinus in diagnosing and managing low-positioned placenta.

## Introduction

A low-positioned placenta refers to a placenta implanted in the lower uterine segment, including placenta previa and low-lying placenta. A placenta previa is characterized by the presence of placental tissue over the internal os of the uterine cervix, whereas a low-lying placenta is diagnosed when the inferior placental edge is located at 1–20 mm from the internal os.^1,2^

The incidence of a low-positioned placenta decreases from 5% in the second trimester to 0.3%–0.9% in the third trimester of pregnancy.^3^ In over 90% of women, a low-positioned placenta will resolve as pregnancy continues.

However, the incidence of this condition has progressively risen in the last decade due to the upward trend of cesarean delivery (CD) and pregnancies conceived by assisted reproductive technology, known risk factors for a low-positioned placenta.^4,5^

Low-positioned placenta is associated with an increased risk of perinatal adverse outcomes,^6^ mostly due to bleeding events and prematurity.^6–8^

Transvaginal sonography (TVS) is the gold-standard technique for accurately diagnosing a low-positioned placenta.^9^ It allows the measurement of the extension of placental tissue beyond the cervix and the distance between the placenta and the internal os (internal-os-distance [IOD]) in cases of placenta previa and low-lying, respectively. TVS also assesses the characteristics of the placental edge, including the presence or absence of marginal sinus.^10^

The marginal sinus is a circumscribed hypoechoic area with venous flow visible in grayscale or with color flow imaging.^10^ It represents discontinuous venous lakes at the margin of the placenta. This area, filled with maternal blood and not indicative of vasa previa,^10^ has been associated with higher odds of antepartum hemorrhage and CD.^11–14^

However, robust data on this topic are lacking. Of note, whether the IOD of a low-lying placenta should be estimated using the leading edge of the placental tissue or of the marginal sinus, if present, remains unclear. This uncertainty impacts both diagnosis and clinical management of pregnancy, as well as decisions on the mode of birth in women with a persistent low-lying placenta^15–17^ due to marginal sinus presence. Thus, providing clearer data on this topic is mandatory to generate evidence supporting clinical practice and enhancing the management of this high-risk obstetric population.

Our study aimed to conduct a systematic review of the literature using a narrative synthesis to evaluate whether marginal sinus presence in women with a low-positioned placenta can impact the risk of antepartum, intrapartum, and postpartum hemorrhage.

## Methods

The systematic review of the literature was carried out following the Preferred Reporting Items for Systematic Reviews and Meta-Analyses guidelines (PRISMA).^18^ The review protocol was established by two investigators (E.C. and S.O.), and prospectively registered with the International prospective register of systematic reviews PROSPERO (registration No. CRD42021265144).

A narrative synthesis approach was followed, according to Popay et al.^19^ and focusing on summarizing and explaining findings through text. A narrative synthesis approach was employed, following a structured framework that included: (1) developing a theory of how the intervention/condition works, why, and for whom; (2) developing a preliminary synthesis of findings of included studies; (3) exploring relationships in the data; and (4) assessing the robustness of the synthesis.

### Search strategy

Relevant studies conducted between 1980 and 2021 were identified by searching the electronic databases PubMed, Scopus, EMBASE, and the Cochrane Library. The first search was conducted on March 12, 2021, and an updated search was carried out on May 19, 2024 to identify additional articles, and no further relevant studies were identified. The search terms strategy is reported in Table 1.

**Table 1.** Detailed Search Strategy for Four Database Searches

*N*°	Database (total four)	Search terms	Results total (*n* = 8140)
1	PubMed	(Marginal sinus placenta AND [birth OR delivery OR hemorrhage OR rupture] OR placenta praevia OR low-lying placenta AND marginal sinus OR hemorrhage).	2491
2	Scopus	TITLE-ABS-KEY (Marginal sinus placenta AND [birth OR delivery OR hemorrhage OR rupture] OR placenta praevia OR low-lying placenta AND marginal sinus OR hemorrhage).	3278
3	EMBASE	TITLE-ABS-KEY (Marginal sinus placenta AND [birth OR delivery OR hemorrhage OR rupture] OR placenta praevia OR low-lying placenta AND marginal sinus OR hemorrhage).	2338
4	Cochrane	(Marginal sinus placenta AND [birth OR delivery OR hemorrhage OR rupture] OR placenta praevia OR low-lying placenta AND marginal sinus OR hemorrhage).	33

Articles were included if they met the following criteria: English or Italian language, systematic reviews, and quantitative primary research studies. Exclusion criteria were: secondary data analyses, case reports, case series, commentaries, letters, and correspondence.

The year 1980 was chosen as the starting date for the systematic review due to significant advancements in ultrasound technology and the establishment of routine 20-week^20^ fetal abnormality screening, including transvaginal scans.

### Study selection

**FIG. 1.**
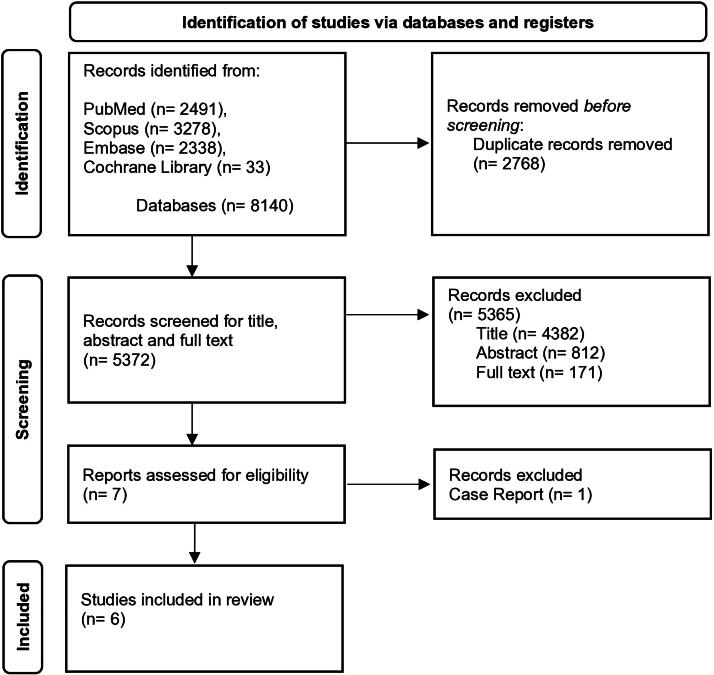
PRISMA flow diagram. PRISMA, Preferred Reporting Items for Systematic Reviews and Meta-Analyses.

The literature search and the inclusion process are detailed according to the PRISMA flow diagram^18^ (Fig. 1). All references retrieved during the systematic search were stored in Mendeley. After duplicate removal, two researchers (E.C. and S.O.) independently screened all titles and abstracts to identify relevant studies according to the eligibility criteria. Abstracts relevant to the search topic were selected for full-text screening. Additional suitable articles were identified by manually searching the references list of selected studies, and their full texts were screened to ensure comprehensive coverage. A list of articles meeting the inclusion criteria was compiled. Two authors (E.C. and S.O.) independently evaluated the studies’ fulfillment of the inclusion criteria. Disagreements were resolved through discussion or consultation with a third reviewer (S.F.) to finalize the selection of relevant studies.

### Data extraction and quality assessment

The review aimed to determine whether the presence of a marginal sinus in women with a low-positioned placenta increases the risk of antepartum, intrapartum, and postpartum hemorrhage regardless of the mode of birth. Data extraction tables were designed accordingly, including authors, study location and design, sample size, study objectives, definition of marginal sinus, key findings, and results. Two reviewers (E.C. and S.O.) independently extracted and summarized the data. Discrepancies were resolved by consensus and, where necessary, a third reviewer was consulted (S.F.).

Study quality was assessed using the Newcastle-Ottawa Scale (NOS)^21^ for cohort studies, a tool to assess the quality of analytical studies and recommended by the Cochrane Collaboration for nonrandomized studies. The NOS evaluates studies based on eight items, categorized into three dimensions, which can be scored: selection (maximum of four stars), comparability (maximum of two stars), and outcome (maximum of three stars).^21^

A second researcher (E.C.) independently verified the quality assessment, with consensus reached.

### Data synthesis

Considering the rarity of the marginal sinus in the context of a low-positioned placenta, all studies were retained without quality-based exclusion to capture potential insights. Quality assessment was applied to critically evaluate evidence strengths and limitations.^10^

Meta-analysis was unsuitable due to high heterogeneity in data. This heterogeneity was both statistical, with standardized effect sizes unavailable for five out of six studies, and clinical, with substantial diversity in population, intervention, comparison, and outcome. Hence, a narrative synthesis was employed to synthesize findings from these diverse studies.

### Narrative Synthesis Element 1: theory development

Accurate assessment of placental location, cervical length, placental edge thickness, and the presence or absence of a marginal sinus is crucial for individualized antenatal care and birth planning.^10^ However, it is unclear whether the placental edge should be defined by the parenchyma or the marginal sinus, if present, leading to inconsistent diagnoses. For instance, a marginal sinus at 5 mm from the internal os, with the placenta parenchyma at 25 mm, might be diagnosed as a low-lying placenta by some obstetricians, while others might consider it normally located with a marginal sinus as a placenta characteristic.^10^

There is limited knowledge about the impact of marginal sinus on the risk of antenatal bleeding, leading to varied clinical management and counseling among obstetricians.

Some women at term may have a persistent low-lying placenta with a marginal sinus. Inconsistency regarding its effect on intrapartum and postpartum bleeding complicates decisions about the mode of birth.

Increasing understanding of the clinical significance of marginal sinus may facilitate professionals to provide high-quality care and avoid unnecessary interventions. It would also improve counseling for women with a low-positioned placenta and marginal sinus, promoting empowerment and informed choices.

## Results

### Narrative Synthesis Element 2: developing a preliminary synthesis

#### Study selection and study characteristics

Search results are summarized in the PRISMA flowchart (Fig. 1). Full-text articles were obtained for 171 studies, with 6 meeting the inclusion criteria.

The characteristics of the included studies are presented in Table 2, the results of the included articles in Table 3, and hemorrhage outcomes categorized by the presence or absence of marginal sinus in Table 4.

**Table 2.** Characteristics of Studies Included in the Systematic Review

Authors, year, country	Study design	Sample	Aim	Definition of marginal sinus
Ishibashi et al. (2018),^23^ Japan	Retrospective cohort study	210 women after 30^+0^ weeks of gestation.	To investigate the clinical significance of marginal sinus placenta previa.	Placental marginal sinus that reaches the internal os and placenta parenchyma might be >2 cm from the internal os (referred to as marginal sinus placenta previa)
Taga et al. (2016),^14^ Japan	Retrospective cohort study	18 women with a low-lying placenta at 36^+0^ weeks of gestation.	To assess the safety of TOL in case of low-lying placenta.	Hypoechoic area with slow and whirl-like blood flow between the placenta edge and the internal os. (If present, the distance between the internal os and the lowest edge of the marginal sinus was regarded as the IOD.)
Ohira et al. (2012),^13^ Japan	Prospective cohort study	49 women with a low-lying placenta after 30^+0^ weeks of gestation.	To examine the significance of resolution of low placentation and how the presence of marginal sinus may predict mode of delivery in women with low-lying placenta.	Echo-free space at the lower edge of the placenta
Hasegawa et al. (2011),^11^ Japan	Retrospective cohort study	182 singleton pregnancies with placenta previa after 20^+0^ weeks of gestation.	To evaluate whether specific ultrasound findings of placenta previa can predict bleeding during pregnancy and emergency CD.	A hypoechoic space showing flow at the placental margin
Hasegawa et al. (2009),^22^ Japan	Retrospective cohort study	127 singleton pregnancies with placenta previa after 20^+0^ weeks of gestation.	To investigate whether maternal history and ultrasound findings can predict massive hemorrhage during CD in patients with placenta previa and abnormally adherent placenta.	A space of low echogenicity showing flow at the placental margin.
Saitoh et al. (2002),^12^ Japan	Prospective cohort study	35 women with placenta previa from 28^+0^ weeks of gestation.	To evaluate whether ultrasound findings may predict massive bleeding during pregnancy and at CD in women with placenta previa and no prior history of CD.	Echo-free space in the marginal areas of the placenta, with an associated turbulent blood flow.

CD, cesarean delivery; IOD, internal-os-distance; TOL, trial of labor.

**Table 3.** Key Findings and Results from the Studies Included in the Systematic Review

Study	Key findings and results	
Ishibashi et al. (2018)^23^	The cohort was divided into three groups: Group A = 27 women with marginal sinus placenta previa. Group B = 75 women with low-lying placenta and marginal placenta previa. Group C = 108 women with partial and total placenta previa. All women underwent planned or emergency CD. Comparison A–B: significant difference in emergency CD (0 vs. 17.3%, *p*-value = 0.02) Comparison A–C: significant difference in antenatal bleeding (11.1% vs. 47.2%, *p* < 0.01), emergency CD (0% vs. 25.9%, *p* < 0.01), intraoperative hemorrhage 1141.8 ± 568.4 vs. 1506.0 ± 918.7 (*p* = 0.03), blood transfusion (0% vs. 19.4%, *p* < 0.01), uterine artery embolization (0% vs. 25.9%, *p* < 0.01), PPH (119.9 ± 133.9 mL vs. 503.4 ± 894.2 mL, *p* < 0.01). Authors concluded that a marginal sinus placenta previa should be considered as a mild type of placenta previa.	
Taga et al. (2016)^14^	TOL = 11 women, 5 had a marginal sinus Planned CD = 7 women, 1 had a marginal sinus. Women who chose TOL were divided into two groups:	
	IOD <10 mm, *N* = 5	IOD ≥10 mm, *N* = 6
	Emergency CD rate = 60%	Emergency CD rate = 0%
	*N* = 3 presence marginal sinus	*N* = 0 presence marginal sinus
	*p* = 0.026	
	Authors concluded that the low-lying placenta hardly resolves if a marginal sinus is present, leading to a higher risk of antepartum hemorrhage and emergency CD due to bleeding.	
Ohira et al. (2012)^13^	Women were divided based on the presence or absence of marginal sinus at 36–37^+0^ weeks of gestation.	
	Presence of marginal sinus *N* = 7	Absence of marginal sinus *N* = 42
	CD rate due to antepartum bleeding = 71.4%	CD rate due to antepartum bleeding = 9.5%
	*p* < 0.01	
	The combination of slow resolution and presence of marginal sinus was associated with CD due to antepartum bleeding.	
	Authors suggested that for women with these characteristics, a planned CD could be considered a reasonable and safer option.	
Hasegawa et al. (2011)^11^	Placenta previa included complete, partial, and marginal placenta previa just prior to CD. All women underwent planned or emergency CD. Antenatal bleeding: 102/182 (56%) Emergency CD due to bleeding: 67/132 (37%) US findings at 20 ^+0^ weeks of gestation (available for 77 women) associated with emergency CD due to bleeding: none US findings at 20 ^+0^ weeks of gestation (available for 77 women) associated with antenatal bleeding: presence of marginal sinus (16% bled with marginal sinus vs. 0% without marginal sinus, *p* < 0.05). Authors’ hypothesis: marginal sinus indicates the retention of maternal blood flow in the intervillous space and decidual tissue, which may collapse occasionally due to uterine contraction, resulting in antepartum hemorrhage.	
Hasegawa et al. (2009)^22^	The diagnosis of placenta previa was confirmed in all patients using transvaginal ultrasound performed after 20 weeks of gestation. Ultrasound findings were obtained within a week before CD. All women underwent planned or emergency CD. Emergency CD: 49 due to bleeding (5 abnormally adherent placenta). The presence of marginal sinus was not associated with either massive antepartum hemorrhage or abnormal placental adherence.	
Saitoh et al. (2002)^12^	Women were divided into three groups: Type A (*N* = 13): two-thirds of the placenta covers the internal os. Type B (*N* = 10): one-third of the placenta covers the internal os. Type C (*N* = 12): echo-free space (marginal sinus) at the placental edge overlying the internal os. All types were further divided into additional groups based on the presence or absence of associated sponge-like echo in the wall of the uterus adjacent to the placental location. All women underwent planned or emergency CD. Massive antepartum bleeding *p* < 0.01: type A = 7.7% (1/13); type B = 10% (1/10); type C = 83.3% (10/12). Preterm birth (30–32 weeks): Five women, four of whom were in type C placenta and had a bleeding event. Authors concluded that the presence of marginal sinus might lead to an increased risk of sudden massive antepartum bleeding.	

Mean ± SD (standard deviation).

ml, milliliters; PPH, post-partum hemorrhage; US, ultrasound scan.

**Table 4.** Antepartum, Intrapartum, and Postpartum Hemorrhage Based on the Presence or Absence of Marginal Sinus

Study	Outcome	Presence of marginal sinus	Absence of marginal sinus without placenta previa	Absence of marginal sinus with placenta previa	Statistical significance
Ishibashi et al. (2018)^23^	Antepartum hemorrhage	11.1% (Group A) (*n* = 27)	N/A	47.2% (Group C) (*n =* 108)	*p* < 0.01
	Intrapartum hemorrhage	1141.8 ± 568.4 mL † (Group A)	N/A	1506.0 ± 918.7 mL (Group C)	*p* = 0.03
	Postpartum hemorrhage	119.9 ± 133.9 mL (Group A)	N/A	503.4 ± 894.2 mL (Group C)	*p* < 0.01
Taga et al. (2016)^14^	Antepartum hemorrhage	60% (*n* = 5)	0% (*n* = 6)	N/A	*p* = 0.026
	Intrapartum hemorrhage	N/A	N/A	N/A	N/A
	Postpartum hemorrhage	N/A	N/A	N/A	N/A
Ohira et al. (2012)^13^	Antepartum hemorrhage	71.4% (*n* = 7)	9.5%(*n* = 42)	N/A	*p* < 0.01
	Intrapartum hemorrhage	N/A	N/A	N/A	N/A
	Postpartum hemorrhage	N/A	N/A	N/A	N/A
Hasegawa et al. (2011)^11^	Antepartum hemorrhage	16% (*n* = 50)	N/A	0% (*n* = 27)	*p* < 0.05
	Intrapartum hemorrhage	N/A	N/A	N/A	N/A
	Postpartum hemorrhage	N/A	N/A	N/A	N/A
Hasegawa et al. (2009)^22^	Antepartum hemorrhage	N/A	N/A	N/A	N/A
	Intrapartum hemorrhage	11.5% (*n* = 26)	13.9% (*n* = 101)	N/A	OR = 0.8 (0.2–3.1)
	Postpartum hemorrhage	N/A	N/A	N/A	N/A
Saitoh et al. (2002)^12^	Antepartum hemorrhage	83.3% (Placenta type C) (*n* = 12)	N/A	7.7% (Placenta type A) (*n* = 13) 10% (Placenta type B) (*n* = 10)	*p* < 0.01
	Intrapartum hemorrhage	N/A	N/A		N/A
	Postpartum hemorrhage	N/A	N/A		N/A

N/A, not applicable; *n*, number of cases; OR, odds ratio.

All studies, conducted between 2002 and 2018 in Japan, varied in design and population.

The systematic review comprises six cohort studies, two prospective^12,13^ and four retrospective,^11,14,22,23^ for a total of 621 women with a low-positioned placenta.

Two studies^13,14^ focused on women with low-lying placenta undergoing a trial of labor. Four studies^11,12,22,23^ involved women with placenta previa^12^ or both previa and low-lying^11,22,23^ who were offered a planned CD.

Four studies^11,13,14,22^ used TVS for placental localization and identification of marginal sinus, two also used color Doppler ultrasound^12^ or MRI.^23^

Three studies^11,12,22^ evaluated ultrasound findings, like marginal sinus presence as predictive factors for bleeding requiring CD for hemorrhage.

The remaining three studies^13,14,23^ focused on women with placenta previa or low-lying, with or without a marginal sinus, investigating maternal and neonatal outcomes such as antenatal bleeding, pregnancy duration, CD rate, hemorrhage, and treatments to control bleeding at birth. Despite their contributions, these studies exhibit weaknesses, including insufficient obstetric history information^12,13^ and unclear statistical adjustments in results analysis.^11–14,23^ Additionally, the clinical implications of their findings are not extensively discussed by the authors.^23^

All the studies considered^11–14,22,23^ the IOD as the distance between the internal os and the lower edge of the marginal sinus. However, only two studies specified that the marginal sinus was involved in diagnosing the type of placenta.^13,14^ Both studies included only women with low-lying placenta undergoing a trial of labor.

Among the reviewed studies, five assessed antenatal hemorrhage as a primary outcome to investigate the impact of marginal sinus. Two studies examined its influence on intrapartum hemorrhage, and one study also evaluated postpartum hemorrhage. Antenatal hemorrhage was defined in three studies, with varying thresholds: <100 mL,^23^ >100 mL,^22^ and ≥300 mL.^13^ Saitoh et al.^12^ reported sudden massive bleeding at >500 mL.

Only one study^23^ defined intrapartum hemorrhage as persistent bleeding >100 mL or uncontrollable uterine contractions.

Postpartum hemorrhage criteria were set at 1500 mL for both vaginal birth and CD^14^ in one study and 2500 mL for CD alone in another.^22^ Ishibashi et al.^23^ defined postpartum hemorrhage as bleeding from the end of CD to 24 hours post-surgery. The remaining studies did not specify the hemorrhage definition.

Blood transfusion was reported in three studies.^12,14,23^

The two studies offering vaginal birth to women with low-lying placenta^13,14^ divided their cohorts to compare different cut-off values of the IODs. Taga et al.^14^ diagnosed low-lying placenta using an IOD ≤20 mm at 36 weeks, while Ohira et al.^13^ used an IOD ≤30 mm for diagnosis but evaluated the mode of birth by comparing groups with IOD <20 mm versus ≥20 mm. Both studies, despite providing valuable data on vaginal birth in women with a low-lying placenta and a marginal sinus, had small sample sizes: *N* = 18^14^ and *N* = 49.^13^

All remaining studies compared groups based on placental features identified during TVS.

The time interval between ultrasound and birth varied from 1 week to several weeks, raising concerns about the consistency of findings. One study did not report the timing of the last scan before CD.^23^

Small sample sizes in all studies limited their generalizability and statistical power.

### Quality assessment of the included studies

Table 5 displays the quality assessment of the included studies. Most studies scored well on the NOS selection criteria, regarding the representativeness of exposed individuals and ascertainment of exposure and outcome. However, four^12–14,23^ cohort studies lacked a nonexposed group, making comparability inapplicable. In the two remaining retrospective studies,^11,22^ authors recruited only women with placenta previa, dividing them into exposed and nonexposed cohorts.

**Table 5.** Quality Assessment of Included Studies According to Newcastle-Ottawa Scale for Cohort Studies

Study	Selection	Comparability	Outcome
Ishibashi et al. (2018)^23^	***	N/A	*
Taga et al. (2017)^14^	***	N/A	*
Ohira et al. (2012)^13^	***	N/A	*
Hasegawa et al. (2011)^11^	****	*	*
Hasegawa et al. (2009)^22^	****	**	*
Saitoh et al. (2002)^12^	***	N/A	*

^a^ N/A, not applicable

In all included studies, outcome assessment was not blinded, and there was no clear reference to secure medical records for confirmation. Blinding was not possible during pregnancy, labor, and birth, as health care professionals needed to be aware of potential risks.

The main weaknesses of these studies included retrospective design, small sample size, and varying gestational ages at diagnosis of low-positioned placenta.

Furthermore, substantial heterogeneity was evident among the six included studies in methodologies, study populations, and outcome measures.

Methodologically, variations exist in techniques for assessing the presence and significance of a marginal sinus in women with a low-positioned placenta, ranging from TVS to MRI. Criteria for defining and categorizing low-positioned placenta, and methods for predicting hemorrhage, also vary across studies. Differences in demographic characteristics such as age, parity, and comorbidities among study populations may influence observed outcomes. Variations in sample sizes and inclusion criteria further contribute to study heterogeneity. Discrepancies in outcome measures, including definitions and timing of antepartum, intrapartum, and postpartum hemorrhage, complicate synthesis and comparison of findings across studies.

This heterogeneity underscores the complexity of studying the association between marginal sinus and hemorrhage risk in low-positioned placentas.

## Discussion

### Narrative Synthesis Element 3: exploring the relationships within and between the studies

All studies used the lower edge of the marginal sinus to diagnose a low-positioned placenta, but a standardized definition was absent. The assessment of bleeding risk in women with this condition remains insufficiently explored. Inadequate data prevent making recommendations on the significance of a marginal sinus and its impact on bleeding risk in low-positioned placenta cases. The evidence from selected studies showed inconsistencies and inconclusive outcomes, highlighting a critical gap in the literature. Further research is needed to clarify this condition and better inform clinical decision-making.

Authors differ in defining placenta previa and low-lying, complicating result comparability. Three studies^11,22,23^ did not report their definitions, and one^13^ used an IOD ≤30 mm for low-lying placenta diagnosis. Some definitions, including “partial” and “marginal” placenta previa, are outdated and inconsistent with current literature^1,2,9^ and with the 2020 Japanese guideline.^24^

Placental location and distance from the internal cervical os are typically diagnosed during the second-trimester ultrasound.^1^ However, four studies^12–14,23^ included women diagnosed later, from 28 to 36 weeks, risking missed cases of asymptomatic resolved low-positioned placenta with a marginal sinus. Although these cases may not have immediate clinical significance, further research is needed to determine whether early detection provides prognostic value in assessing bleeding risk. Notably, a recent study found that women with resolved low placentation still faced a higher risk of postpartum hemorrhage and related complications.^25^

Four studies^11–14^ observed a significant association between marginal sinus presence and antenatal bleeding. Ohira et al.^13^ found that marginal sinus alongside slow placental migration increased antepartum bleeding frequency. Taga et al.^14^ concluded that women with a low-lying placenta and marginal sinus are more likely to bleed during pregnancy, suggesting that limited placental upward movement toward the third trimester’s end elevates the risk of antepartum bleeding and emergency CD.^26^

In three out of five cases with an IOD <10 mm, marginal sinus, and slow placental migration, uncontrollable antenatal bleeding occurred. A shorter IOD is known to increase the risk of antepartum hemorrhage,^26^ making it challenging to pinpoint which of these three elements contributes most significantly to bleeding.

Although third-trimester antepartum blood loss is a significant risk factor for intrapartum bleeding requiring emergency CD,^16,26^ none of the reviewed studies addressed this topic.

Ohira et al.^13^ and Taga et al.^14^ were the only studies in this review offering a vaginal birth to participants, allowing for comparison in terms of postpartum hemorrhage. However, neither assessed the association between marginal sinus presence and postpartum hemorrhage. Taga et al.^14^ observed higher rates of intrapartum hemorrhage and blood transfusions in the planned CD group compared with the vaginal delivery group, consistent with previous evidence.^15,17,27,28^

Ohira et al.^13^ speculated that marginal sinus rupture near the internal os might cause bleeding, aligning with Ryo and colleagues’ hypothesis^29^ and with prior publications identifying such ruptures as a common cause of antepartum bleeding.^30,31^

Saitoh et al.^12^ demonstrated a higher risk of massive antepartum hemorrhage with a marginal sinus overlying the internal os, contrasting with Hasegawa et al.^22^ and Ishibashi et al.^23^ Hasegawa and colleagues^22^ observed no correlation between the marginal sinus and significant bleeding during pregnancy or CD. However, in this study, transvaginal ultrasound was performed only after 20 weeks of gestation, at which point the marginal sinus may have already resolved and become undetectable. Interestingly, in a subsequent study,^11^ the same authors reported that marginal sinus at the 20-week scan might be associated with minor antenatal bleeding episodes. Notably, ultrasound findings were available for only 42.3% of women (*N* = 77/182).

Ishibashi et al.^23^ found that placentas with a marginal sinus bleed significantly less than other types of placenta previa or low-lying, during pregnancy, birth, and postpartum. The study defined this as “marginal sinus placenta previa” and considered it a mild type of placenta previa. Despite lacking a clear explanation, it is plausible that the authors considered the marginal sinus as the IOD, suggesting planning a CD in these cases to avoid bleeding.

None of the remaining studies^11–14^ investigated the link between marginal sinus and intrapartum or postpartum hemorrhage.

Among the two studies on mode of birth in women with low-lying placenta,^13,14^ Ohira et al. diagnosed low-placentation with an IOD ≤30 mm but compared birth modes between IOD <20 mm and ≥20 mm. They found higher CD rates in women with a marginal sinus, suggesting a potential link with antepartum bleeding. Among the seven women with a marginal sinus, five also had a slow migration and underwent CD. This suggests that the combination of marginal sinus and slow placental migration rate might predict the mode of birth, consistent with other research linking low-positioned placenta and slow migration to higher CD rates.^32,33^

Taga et al.^14^ recommended a trial of labor for all low-lying placenta cases except those with a marginal sinus, due to a higher risk of antepartum bleeding requiring emergency CD. In their study, five women showed a marginal sinus and slow placental migration; three had an emergency CD due to bleeding, whereas two gave birth vaginally.

Ishibashi et al.^23^ enrolled women with a low-lying placenta but did not offer a trial of labor. Interestingly, authors noted a lower rate of emergency CD among women with a marginal sinus, possibly due to less bleeding when an abruptio of a marginal sinus not overlying the internal os occurs, compared with an abruptio involving a marginal sinus or placental tissue overlying the internal os.

The remaining studies in this systematic review recruited women with placenta previa, recommending planned CD as the mode of birth.

Bleeding risk in women with a low-positioned placenta and marginal sinus has been inadequately evaluated so far.

Low-positioned placenta, particularly placenta previa, is a leading cause of pregnancy and childbirth bleeding, contributing to maternal and fetal life-threatening events.^6^ Therefore, accurately predicting bleeding before its occurrence is crucial for planning appropriate obstetric care.

TVS is the current gold standard for identifying placental location.^2^ However, there’s uncertainty about whether the parenchyma or the marginal sinus should define the placental edge in diagnosing a low-positioned placenta. Despite suggestions in the studies included in this review that a marginal sinus reaching the internal os should define a low-positioned placenta, most studies on the topic do not specify its significance regarding bleeding risk and mode of birth.

The included studies lack conclusive evidence due to insufficient power and inconsistent results. Therefore, a clear recommendation cannot be made at this time. A prospective multicenter study^34^ is ongoing to gather additional evidence, which may offer valuable insights soon. Identifying ultrasound findings associated with the risk of antepartum, intrapartum, or postpartum hemorrhage is of utmost clinical relevance in current obstetric practice.

Finally, to improve outcomes-based research, we suggest the adoption of standardized definitions for low-lying placenta in accordance with international guidelines^24^ and the consistent reporting of marginal sinus when detectable.

We hope this systematic review lays the groundwork for future research to enhance reliability and validity in this field.

### Narrative Synthesis Element 4: assessment of the robustness of the synthesis

#### Strength and limitations

This is the first narrative synthesis systematic review on this topic, employing a rigorous search strategy, quality assessment of studies, and systematic analysis ensuring transparency.

The assessment of the robustness of this narrative synthesis systematic review pivots on the quantity and quality of evidence, alongside bias-minimizing methods.

Based on a PubMed search using “placenta” and “marginal sinus” as keywords, only 51 articles were found, with approximately 60% published in the 1950s and 1960s. Thus, inclusion and exclusion criteria were established to minimize the bias of missing relevant studies.

Since all studies were based in Japan and conducted by Japanese authors, this may introduce selection bias due to the homogeneity of obstetric care and birth culture.

The Japan Society of Obstetrics and Gynecology recommends plan CD by the end of 38th weeks gestation for women with a low-lying placenta (IOD <20 mm), according to the 2020 guidelines edition published in 2024.^24^ The lack of blinding and adherence to clinical guidelines may bias results, particularly favoring planned CD for women with low-lying placenta.

Additionally, small sample sizes, especially in studies offering a trial of labor to women with low-lying placenta (*N* = 18^14^; *N* = 49^13^) limit the generalizability and robustness of findings.

Furthermore, the predominance of retrospective studies increases the risk of selection bias, incomplete data, and difficulties in distinguishing between retrospective and prospective datasets. Prospective cohort studies are challenging due to the rarity of encountering a marginal sinus with a low-positioned placenta. Acknowledging these challenges is crucial for improving result reliability and overall evidence quality.

The narrative synthesis systematically described the study aims, objectives, and hemorrhage-related outcomes. Quality assessment tools were applied to identify biases in study design, conduct, and reporting. Discrepancies in definitions and outcome measurements, especially regarding hemorrhage, introduce heterogeneity and complicate comparisons. Inconsistent terminology for classifying low-positioned placenta also hinders differentiation of outcomes between placenta previa and low-lying, limiting valuable information. Moreover, a detailed analysis of concurrent medical and obstetric factors influencing bleeding outcomes was lacking.

Despite these limitations, the narrative synthesis used a rigorous search strategy and transparent quality assessment of included studies, thereby enhancing its reliability.

Overall, the systematic review offers interesting insights into the association between marginal sinus and hemorrhage and mode of birth. However, limitations in the amount and quality of evidence and potential biases in the included studies highlight the need for further research to establish more robust evidence on this topic.

## Abbreviations used

ART: Assisted Reproductive Technology
CD: Cesarean Delivery
IOD: Internal-Os Distance
TVS: Transvaginal Sonography
